# Puerperal Convulsions

**Published:** 1881-05-20

**Authors:** T. S. Lallerstedt

**Affiliations:** Georgia


### PUERPERAL CONVULSIONS.

BY T. S. LALLERSTEDT, M.D., OF GEORGIA.

What causes puerperal convulsions? After carefully examining a few works on obstetrics I find that the prime cause is albumen in the urine. I claim that it is irritation of either the womb or external genital organs; for it is a well settled fact that more women have convulsions with their first confinement than those who have borne children before. And why irritation? Because as soon as you either remove the child or give something to quiet the nervous system, then you have no more convulsions.

Prof. Meigs, among the oldest American writers on obstetrics, says: "The pregnant woman who reaches her term and falls into labor without having suffered from œdema or other results of pressure will rarely be found to have an attack of puerperal convulsions, while every woman who has swelled feet and legs in her gestation; every woman whose urine, on being tested, exhibits the presence of albumen; all those who complain of headache, transient seizures with amaurosis, tinnitus aurium, deafness, convulsive twitchings, red and tumid hands and fingers, and all in whose urine casts of tubuli uriniferi are discovered by the microscope, all such people should be regarded as in danger of convulsions."

On pages 240 and 241 will be found about the same. In Churchill's System of Midwifery, commencing on page 450 and extending to 453, a great deal about what may be the cause, the principal causes I condense: Sympathy of the brain with the irritation of some different and often distant organ, it may be the uterus, the stomach or bowels; intemperance in eating or drinking; mental emotions and fright; accumulation of blood in the head; atmospheric influence.

I have not given all of his causes, but those of most importance.

"Colombat on Diseases of Females," we find on pages 632 and 633: "The causes of eclampsia ought to be divided into predisponent and occasional causes. The first in order of these conditions is undoubtedly the primaparous state, for, according to a statement made by Dr. Collins, of Dublin, there were seventy-five primaparous women in eighty-five cases of convulsive attack during pregnancy and lying-in. Women in their first pregnancy are more liable to eclampsia, only because in them the uterus enjoys a higher degree of susceptibility, and the labor, moreover, is longer and more painful. Among the predisponent causes of the disorder we ought also to class the distension of the womb by twins or by an unusual quantity of water which almost always coincides with a serous diathesis and considerable infiltration of the inferior extremities. The sanguine temperament and particularly the lymphatic temperament, with general and partial œdema of the cellular tissue, are rationally by many authors regarded as condittons essential to the production of eclampsia."

In Cazeaux's "Midwifery," are mentioned the following causes: "Albumen in urine, organic lesion of kidneys, extreme distension of uterus, first pregnancy, alteration in quantity or quality of the blood, the irritation produced by distension of the intestinal canal, indigestible food in stomach, irritation of the walls of the bladder produced by its extreme distension with urine."

Prof. Meigs: "Doctrine of swollen legs will not do, for we have often seen and confined women whose extremities were so much swollen that they were confined either to a chair or bed, and yet had no convulsions."

Churchill's views as to a distended stomach, uterus or bowels producing convulsions will not hold, for frequently the uterus becomes so large that the waters are expelled several days before labor sets in, and the stomach almost invariably relieves itself by vomiting, and as a general rule women keep their bowels well open.

Colombat appears to think that primaparous women are more liable to convulsions, and says that "labor is longer and more painful." I don't think that will do, for young women, in my experience, get through about as quick as those who have borne children before. Cazeaux's views are more nearly allied to what I believe.

My experience with puerperal convulsions is not extensive, but close observation has not inclined me to the albumen theory. I have only seen five cases of puerperal convulsions, but one of which I had charge of at the start. I say the disease is from womb irritation; why? For the reason that, unless it was reflex irritation, we would have no convulsions.

The first case I ever saw was a woman 35 years of age and a primapara; the head of child was large, and as it passed out of vulva, she had a convulsion. As soon as she could swallow I gave 30 grains of sulphate of cinchonia, and in about half-hour I found that the pupils of her eyes began to dilate and I gave 20 grains more. In about one hour I noticed the same symptoms and gave 15 grains more, and that was the last of them. Was not the convulsion caused by the irritation of external genitals from the passage of the head?

The second case I saw died in a few hours after I was called to see her. Nothing that I could do gave her any relief. The situation of her womb was as low down as it could be within the pelvic bones. The convulsions were caused, in my belief, by pressure.

The third case, when I saw her, had been having convulsions for eight hours when I arrived in consultation. Nothing but cold water to head gave her any relief. The womb was as low as it could get, and the water had escaped long before. Mouth of uterus dilated to the size of a quarter dollar. We dilated the womb and used every means to deliver her, and finally performed craniotomy. We couid not turn and deliver because the womb was so contracted down it was impossible to pass in the hand. After she was delivered, convulsions ceased, and she soon recoverd but died in next labor without any convulsion, from exhaustion, as I learned.

The fourth case was a negro woman who had had convulsions for 12 hours when I arrived at her home. Her medical attendant had given her up, and left for his home. The situation of the womb was low down, and mouth undilated, so as you could, by hard pressure only, get your index finger into it. The mouth felt like that of a sucker fish. I did not attempt to deliver her, but first bled her profusely—which did no good. I then gave chloroform with poor success. I then gave hypodermically 9 drops veratrum viride and 20 drops fluid extract gelseminum in the left arm. She had no more convulsions. I remained with her until 10 o'clock, and she was restless. I gave ¼ gr. morphine in the arm. On leaving her, the pulse in left arm was almost imperceptible, while right was sixty beats per minute, and strong. I called next morning about 7 o'clock to see her. She was sleeping quietly and had all night. The pulse in left arm was a little stronger and fuller; right, about normal. I left large doses of quinine and Dover's powders for her to take every four hours until my return. She had her child two days afterwards, and so easily it did not wake her up out of a sleep. She soon recovered her usual health.

The fifth case happened recently in my practice. She was taken on morning of 28th December last. The labor was slow and tedious. About 7 o'clock I made an examination and attempted during a pain by placing my hand above the head to hold it down, as in my judgment it was too high above the symphysis pubis, and by that means to guide it into the outlet; but as soon as the pains grew strong she went into convulsions. I soon delivered her with forceps and gave her by mouth veratrum viride and gelseminum. In about one hour and a half she had another convulsion; I repeated above dose. At

12 o'clock she had another convulsion; I then gave 8 drops veratrum viride and 15 drops of the gelseminum. She had one very light convulsion at 2 o'clock. I remained until five o'clock. She recovered rapidly. In her case, convulsions were caused, I think, by the extraordinary pressure.

The third and fourth cases mentioned were clearly the result of reflex irritation from pressure on nerves in pelvic region.

On page 899 of the *American Journal of Obstetrics,* is reported a case of cataleptic convulsions, how cured, etc. I quote as follows: "After the introduction of Sims speculum first demonstrated the existence of a laceration on the left side of the cervix; then taking a probe and placing the point of it in the angle of the laceration. *Slight pressure provoked convulsions, etc., etc.*"

Now, if this lady had been pregnant and at full period, had convulsions, what would they have been called? They would have undoubtedly been called puerperal convulsions. I bring this case up because it gives positive demonstration that pressure will cause convulsions. I, therefore, say, that puerperal convulsions are caused by reflex irritation. I hope that those of the Profession whose field of observation have been more extended than mine will give us their views.

